# Alleviating insomnia should decrease the risk of irritable bowel syndrome: Evidence from Mendelian randomization

**DOI:** 10.3389/fphar.2022.900788

**Published:** 2022-08-16

**Authors:** Wenzhao Bao, Li Qi, Yin Bao, Sai Wang, Wei Li

**Affiliations:** ^1^ Department of Anesthesiology, The Affiliated Hospital of Inner Mongolia University for the Nationalities, Tongliao, China; ^2^ Department of Otorhinolaryngology, The First Hospital of China Medical University, Shenyang, China

**Keywords:** sleep disorder, insomnia, irritable bowel syndrome, Mendelian randomization, causal inference

## Abstract

**Background:** Associations have been reported between sleep and irritable bowel syndrome (IBS). However, whether there exists a causation between them is still unknown.

**Methods:** We employed the Mendelian randomization (MR) design to explore the causal relationship between sleep and IBS. All genetic associations with sleep-related traits reached genome-wide significance (*p*-value < 5 × 10-8). The genetic associations with IBS were obtained from two independent large genome-wide association studies (GWAS), where non-FinnGen GWAS was in the discovery stage and FinnGen GWAS was in the validation stage. Primarily, the inverse-variance weighted method was employed to estimate the causal effects, and a meta-analysis was performed to combine the MR estimates.

**Results:** In the discovery, we observed that genetic liability to the “morning” chronotype could lower the risk of IBS [OR = 0.81 (0.76, 0.86)]. Also, the genetic liability to insomnia can increase the risk of IBS [OR = 2.86 (1.94, 4.23)] and such causation was supported by short sleep duration. In the validation stage, only insomnia displayed statistical significance [OR = 2.22 (1.09, 4.51)]. The meta-analysis suggested two genetically-determined sleep exposures can increase the risk of IBS, including insomnia [OR = 2.70 (1.92, 3.80)] and short sleep duration [OR = 2.46 (1.25, 4.86)]. Furthermore, the multivariable MR analysis suggested insomnia is an independent risk factor for IBS after adjusting for chronotype [OR = 2.32 (1.57, 3.43)] and short sleep duration [OR = 1.45 (1.13, 1.85)]. IBS cannot increase the risk of insomnia in the reverse MR analysis.

**Conclusion:** Genetic susceptibility to insomnia can increase the risk of IBS, and improving sleep quality, especially targeting insomnia, can help to prevent IBS.

## Introduction

Irritable bowel syndrome (IBS) is a chronic functional disorder of the lower gastrointestinal tract with symptoms including abdominal pain, constipation, or diarrhea. There is a higher prevalence of IBS where up to 20% of the population is affected in their lives (Ford et al., 2020). The patients are mainly young and middle-aged. However, its etiology and pathogenesis are still unclear, which may relate to genetics, intestinal motility, the mental system, psychological stress, infection, and chronic inflammation. In addition to drug therapy, several therapies can alleviate the symptoms of IBS, including dietary adjustment, and psychological and behavioral therapy (Vasant et al., 2021). Therefore, a clear understanding of its risk factors is conducive to identifying the mechanism of IBS considering there is no clear and effective therapeutic schedule for IBS and the disease-associated burden on the health service is heavy (Ford et al., 2020).

Sleep occupies almost 1/3 of our lives. Good quality sleep is essential to maintain physical and mental health (Stern, 2021). Sleep plays an important role in human growth and development, metabolism, and memory function, besides, cardiovascular diseases and metabolic diseases will also be affected by sleep disorders (Ai and Dai, 2018; Ai et al., 2021; Song et al., 2021). A recent study implicated frequent daytime napping should have deleterious effects on cardiometabolic traits and daytime napping shared genetic loci with both cardiometabolic and neurodegenerative traits (Dashti et al., 2021). In addition, insomnia has been identified as an important predictor of depression, anxiety, and neuropathy (Lane et al., 2019). Sleep disorders have been reported to be associated with IBS and may account for a proportion of its pathogenesis (Vege et al., 2004; Wang et al., 2018). For instance, the alterations in day-night rhythms could upregulate circulating cytokines *via* triggering sleep disruption, increasing the risk of IBS (Orr et al., 2020). Furthermore, obstructive sleep apnea, usually accompanied by snoring, was also associated with an increased risk of IBS (Rotem et al., 2003; Orr et al., 2020). However, the causal relationship between different types of sleep disorders and IBS remains poorly understood.

Mendelian randomization (MR) studies used genetic variants to verify causal associations between the predefined exposure and outcome (Emdin et al., 2017). This approach is based on a simple principle: if an exposure trait is a risk factor for a kind of disease, then the locus of genetic variation representing that exposure will also be associated with the disease *via* the exposure. Due to the principle of random allocation during meiosis, gene variant loci are usually inherited stably and independently of disease status and environment. Based on these assumptions, the MR design is thus similar to randomized controlled trials in clinical practice and is easier to avoid confounding factors and reverse causation to some extent.

In this study, we used the two-sample MR to explore the causal association between eight different sleep disorders and IBS, hoping to clarify the correlation between sleep disorders and IBS and provide novel insights into the prevention and treatment of IBS.

## Methods

### Data source description

A total of eight sleep-related exposures were included in this study, namely, insomnia, sleep duration (continuous variable), long sleep duration (binary variable), short sleep duration (binary variable), chronotype, “morning” person, daytime nap, and snoring (Table 1). The GWAS of insomnia was analyzed in 453,379 European participants (345,022 cases and 108,357 controls) (Lane et al., 2019). Sleep duration (446,118 Europeans), longer sleep duration (34,184 cases and 305,742 controls), and shorter sleep duration (106,192 cases and 305,742 controls) were extracted from the same GWAS where long sleep duration was defined to be more than 9 h and short sleep duration was less than 6 h (Dashti et al., 2019). The GWAS of chronotype was performed in 449,734 European ancestry individuals and it is a categorical variable, including “Definitely a “morning” person” (coded as 2), “More a “morning” than “evening” person” (coded as 1), “More an “evening” than a “morning” person” (coded as -1), “Definitely an “evening” person” (coded as −2), and “Do not know” or “Prefer not to answer” (coded as 0) (Jones et al., 2019). Similarly, the “morning person” is a binary variable, including 252,287 cases (“Definitely a “morning” person” and “More a “morning” than “evening” person”) and 150,908 controls (“Definitely an “evening” person” and “More an “evening” than a “morning” person”) (Jones et al., 2019). Genetic variants of daytime nap were obtained from a GWAS consisting of 452,633 European individuals, and the phenotype was treated as a continuous variable based on the frequency (Dashti et al., 2021). We extracted genetic variants of snoring from a GWAS consisting of 152,302 cases and 256,015 British ancestry controls (Campos et al., 2020).

**TABLE 1:** Descriptive information of sleep-related data.

Exposure	Ancestry	Sample size	Covariates	NSNP	R2 (%)	F	PMID
Insomnia	European	345,022 cases and 108,357 controls	age, sex, 10 genetic principal components, and genotyping array	44	0.43	44.09	30,804,566
Sleep duration	European	446,118	age, sex, 10 genetic principal components, genotyping array and genetic relatedness matrix	77	0.72	41.72	30,846,698
Long sleep duration	European	34,184 cases and 305,742 controls	age, sex, 10 genetic principal components, genotyping array and genetic relatedness matrix	12	0.14	40.39	30,846,698
Short sleep duration	European	106,192 cases and 305,742 controls	age, sex, 10 genetic principal components, genotyping array and genetic relatedness matrix	25	0.24	40.46	30,846,698
Chronotype	European	449,734	age, sex, study center and genotyping array	196	2.04	47.73	30,696,823
Morning person	European	252,287 cases and 150,908 controls	age, sex, study center and genotyping array	147	1.64	45.73	30,696,823
Daytime nap	European	452,633	age, sex, 10 genetic principal components, genotyping array and genetic correlation matrix	114	1.25	50.27	33,568,662
Snoring	European	152,302 cases and 256,015	age, sex, genotyping array and the first 20 genetic principal components	43	0.41	39.40	32,060,260

Two GWASs of IBS were used, where the discovery set encompassed 53,400 European cases and 433,201 European controls (Eijsbouts et al., 2021) and the validation set was from the FinnGen consortium, including 4,605 cases and 182,423 controls (finn-b-K11_IBS, https://www.finngen.fi/fi). The IBS cases from UKB should meet at least one of the following four conditions (Ford et al., 2020): digestive health questionnaire (DHQ) Rome III: meet Rome III symptom criteria for IBS diagnosis without other diagnostic explanations for these symptoms (Vasant et al., 2021); DHQ “self-report”: answered “yes” to the question “Have you ever been diagnosed with IBS?‘ (Stern, 2021); Unprompted ‘self-report”: self-reported IBS diagnosis in response to the question “Has a doctor ever told you that you have any serious medical conditions?” (Song et al., 2021); the international code of disease version 10 (ICD-10); with hospital episode statistics indicating being admitted to a hospital due to IBS as the main or secondary ICD-10 diagnosis. The cases from FinnGen all met the ICD-10 standard. The former was adjusted for sex, age, and sex*age interaction; age^2^, sex*age^2^ interaction; and the first 20 genetic principal components. The FinnGen GWAS was adjusted for sex, age, the first 10 genetic principal components, genotyping batch, and genetic relatedness matrix. Genomic control has been applied to all these GWASs.

### Mendelian randomization design

**FIGURE 1:**
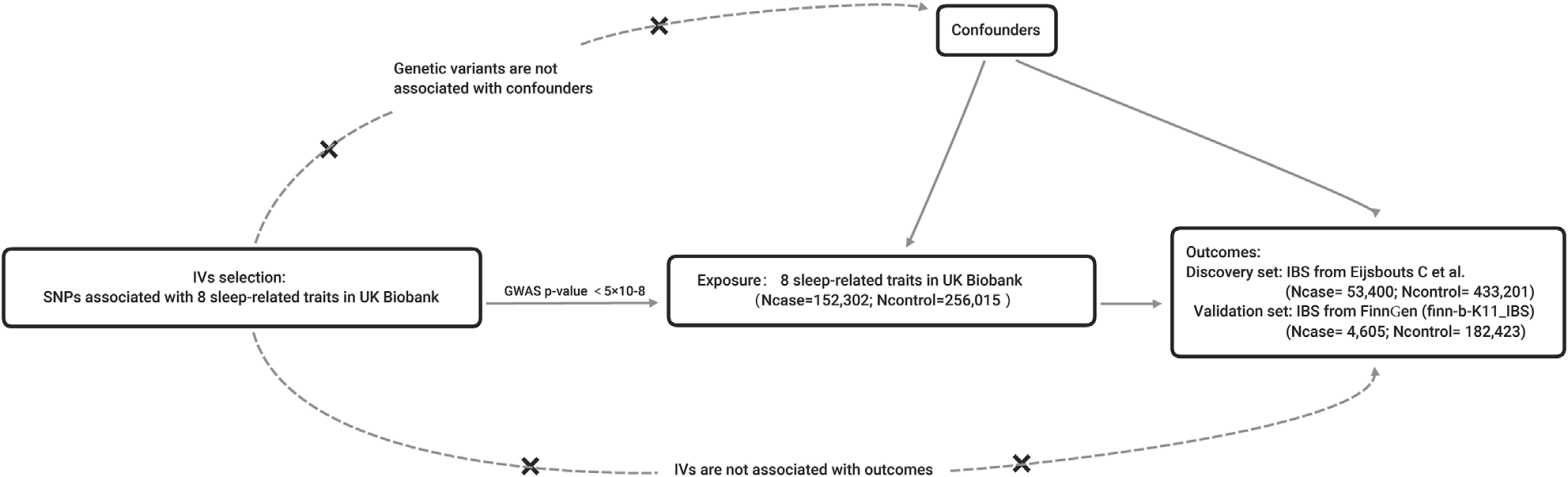
The principles and main design of this Mendelian randomization study. Notes: IV = instrumental variable; SNP = single nucleotide polymorphism; Ncase = the number of cases; Ncontrol = the number of controls.

The MR should be established based on three principal assumptions (Ford et al., 2020): relevance: the IVs should be closely associated with the exposure (Vasant et al., 2021); independence: the IVs should not be associated with any potential confounders that might influence the exposure or outcome (Stern, 2021); exclusion-restriction: the IVs can only affect the outcome *via* the way of exposure and there were no other alternative ways (Emdin et al., 2017) (Figure 1). Other additional assumptions should also be satisfied, such as linearity and no interaction between exposure and mediators.

The most important step in MR analysis is selecting appropriate genetic variants as instruments. Genetic variants, usually common single nucleotide polymorphism (SNP) with a minor allele frequency of more than 0.01, were selected as instrumental variables (IVs) if reaching the genome-wide significance (GWAS *p*-value < 5 × 10^−8^), and then they were clumped based on linkage disequilibrium (*R* ^2^ = 0.01) and distance (10,000 kb). The IBS GWAS with the largest sample size was treated as the discovery set, and the IBS GWAS of FinnGen was the validation set. In the discovery stage, we estimated each exposure’s effect on the IBS using a two-sample MR method, and the same method was applied to the validation stage. Only the exposure significant in both discovery and validation stages was assumed to be the causal risk factor.

Furthermore, the results from both the discovery and validation stages were combined using a meta-analysis method. Considering that sleep-related traits were closely correlated, a multivariable MR in the two-sample summary data setting was adopted to explain the independent causal effect as well (Sanderson et al., 2019). Also, a reverse MR was considered to estimate the reverse causation.

### Statistical methods

The F statistic was calculated to assess the IV validity and assess weak instrument bias. Also, the MR Steiger test was performed to guarantee that each IV explained more variance in exposure than that of outcome (Hemani et al., 2017). In MR estimation, the inverse-variance weighted (IVW) method was utilized as the primary analysis, and two complementary methods were adopted as well, including the MR-Egger and weighted median methods (Bowden et al., 2016; Burgess and Thompson, 2017). It should be noted that IVW can obtain an unbiased result only when all IVs are valid, however, MR-Egger and weighted median methods can estimate the causal effect assuming some IVs are invalid. Additionally, the IVW method was also applied in the multivariable MR analysis (Sanderson et al., 2019). In the discovery stage, the false discovery rate (FDR) method was used to control the false-positive results (FDR <0.05).

### Sensitivity analysis

Several methods have been applied to perform sensitivity analyses, including Cochrane’s Q value for heterogeneity assessment, MR-PRESSO for outliers, and horizontal pleiotropy detection and leave-one-out sensitivity analyses (Verbanck et al., 2018). If there was heterogeneity, the random-effects model for IVW was adopted. The MR-PRESSO method performed the outlier and distortion tests to detect outliers that might bias the results, and the outliers were eradicated from further analyses. The leave-one-out analysis is another standard method for sensitivity analysis that recalibrates the results after removing SNPs one by one, and the SNP should be a driver for the MR estimates if the results change significantly after removal.

### Power calculation and bias assessment

The power calculation was performed using mRnd (https://cnsgenomics.shinyapps.io/mRnd/). The bias caused by sample overlap was assessed by the method proposed by Burgess et al. (https://sb452.shinyapps.io/overlap/).

All statistical analyses and data visualization were performed using R programming software 4.1.2 and the used R packages included “TwoSampleMR”, “MRPRESSO”, “meta”, and “forestplot”.

## Results

### IV description and validity

The number of IVs for each exposure varied from 12 (long sleep duration) to 196 (chronotype). The F statistic for each SNP was greater than the empirical threshold of 10 (Chen et al., 2022) and the overall F statistic for each exposure was larger than 10 as well, suggesting the results were less likely to be biased by the weak instruments. The MR Steiger test indicated that each IV explained more variance in exposure than that of the outcome, meaning the results might not be biased by the reverse causation.

### Causal associations between sleep and IBS in the discovery stage

**FIGURE 2:**
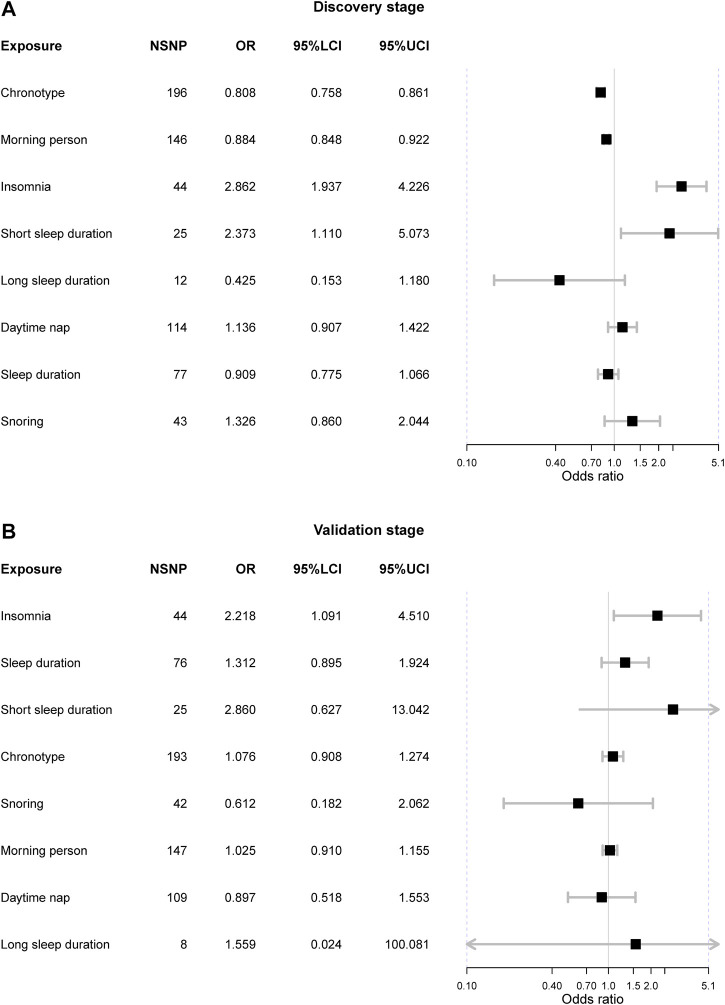
The Mendelian randomization results from both discovery and validation stages. Notes: NSNP = the number of single nucleotide polymorphism; OR = odds ratio; 95%LCI = the lower limit of 95% confidence interval; 95%UCI = the upper limit of 95% confidence interval.

In the discovery stage, three exposures were causally associated with IBS after FDR correction (Figure 2). Initially, we observed genetic liability to the “morning” chronotype could lower the risk of IBS [OR = 0.81 (0.76, 0.86), *p*-value = 3.97 × 10^−11^, FDR = 3.18 × 10^−10^] and it was also supported by it that genetic predisposition to “morning person” tended to reduce the risk of IBS [OR = 0.88 (0.85, 0.92), *p*-value = 6.66 × 10^−9^, FDR = 2.66 × 10^−8^]. Besides, the genetic liability to insomnia can increase the risk of IBS [OR = 2.86 (1.94, 4.23), *p*-value = 1.26 × 10^−7^, FDR = 3.36 × 10^−7^] and such causation was evidenced by the fact that the short sleep duration can suggestively increase IBS as well [OR = 2.37 (1.11, 5.07), *p*-value = 0.026, FDR = 0.05].

The horizontal pleiotropy test based on the MR-Egger intercept indicated that there was no horizontal pleiotropy (MR-Egger intercept *p*-value > 0.05), suggesting that there was no need to correct the intercept in the MR analysis. However, Cochrane’s Q value suggested there existed substantial heterogeneity in all exposure-outcome associations (Q *p*-value < 0.05) except the association between long sleep duration and IBS. The causal relationship between chronotype, “morning” person, insomnia, and IBS remained significant in the weighted median methods (Table 2). Furthermore, the MR-PRESSO method detected outliers in six exposure-outcome associations, and these exposures included chronotype, insomnia, short sleep duration, snoring, sleep duration, and daytime nap. After removal of outliers, the previous observed significant associations were also significant, including chronotype (corrected *p*-value = 6.49 × 10^−11^) and insomnia (corrected *p*-value = 2.32 × 10^−7^) (Table 2). Leave-one-out sensitivity analysis did not detect any other outliers.

**TABLE 2:** Mendelian randomization results from weighted median and MR-PRESSO methods.

Exposure	Stage	NSNP	Weighted median				MR-PRESSO				P_heterogeneity_	P_pleiotropy_
			OR	95%LCI	95%UCI	*p*	OR	95%LCI	95%UCI	*p*		
Chronotype	Discovery	196	0.81	0.74	0.88	0.00	0.81	0.76	0.86	0.00	0.00	0.20
Insomnia	Discovery	44	2.76	1.86	4.09	0.00	2.60	1.81	3.74	0.00	0.00	0.20
Long sleep duration	Discovery	12	0.36	0.09	1.39	0.14	------	------	------	------	0.86	0.41
Morning person	Discovery	146	0.89	0.84	0.94	0.00	------	------	------	------	0.02	0.43
Daytime nap	Discovery	114	1.12	0.87	1.43	0.37	1.15	0.94	1.41	0.17	0.00	0.07
Short sleep duration	Discovery	25	1.95	0.92	4.13	0.08	1.96	0.98	3.93	0.06	0.00	0.28
Sleep duration	Discovery	77	0.92	0.77	1.10	0.36	0.91	0.78	1.06	0.22	0.00	0.97
Snoring	Discovery	43	1.51	0.94	2.43	0.09	1.60	1.10	2.32	0.01	0.00	0.48
Chronotype	Validation	193	0.97	0.76	1.26	0.84	------	------	------	------	0.50	0.45
Insomnia	Validation	44	4.09	1.36	12.30	0.01	------	------	------	------	0.45	0.25
Long sleep duration	Validation	8	0.37	0.00	45.03	0.69	------	------	------	------	0.27	0.79
Morning person	Validation	147	1.05	0.89	1.25	0.56	------	------	------	------	0.26	0.28
Daytime nap	Validation	109	0.97	0.45	2.10	0.95	------	------	------	------	0.07	0.82
Short sleep duration	Validation	25	3.46	0.41	29.39	0.26	------	------	------	------	0.92	0.50
Sleep duration	Validation	76	1.11	0.63	1.95	0.71	------	------	------	------	0.11	0.60
Snoring	Validation	42	0.51	0.11	2.35	0.39	------	------	------	------	0.03	0.94

NSNP, the number of single nucleotide polymorphism; OR, odds ratio; 95%LCI, the lower limit of 95% confidence interval; 95%UCI, the upper limit of 95% confidence interval; P = the *p*-value of OR; P_heterogeneity_ = the *p*-value of heterogeneity test; P_pleiotropy_ = the *p*-value of horizontal pleiotropy test.

### Causal associations between sleep and IBS in the validation stage

In the validation stage, only the insomnia indicated statistical significance [OR = 2.22 (1.09, 4.51), *p*-value = 0.028]. Also, such a result was supported by the weighted median method [OR = 4.09 (1.36, 12.30), *p*-value = 0.012]. Neither heterogeneity nor horizontal pleiotropy was detected in the analysis (Cochrane’s Q *p*-value > 0.05 and MR-Egger intercept *p*-value > 0.05). The MR-PRESSO method did not find outliers that might distort the results, and the leave-one-out sensitivity analysis did not find IV that could drive the results.

We did not observe the causal association between chronotype and IBS [OR = 1.08 (0.91, 1.27), *p*-value = 0.397], nor did the “morning” person [OR = 1.03 (0.91, 1.16), *p*-value = 0.682]. Also, there was no heterogeneity or horizontal pleiotropy.

### Meta-analysis of MR results from the discovery and validation stage

**FIGURE 3:**
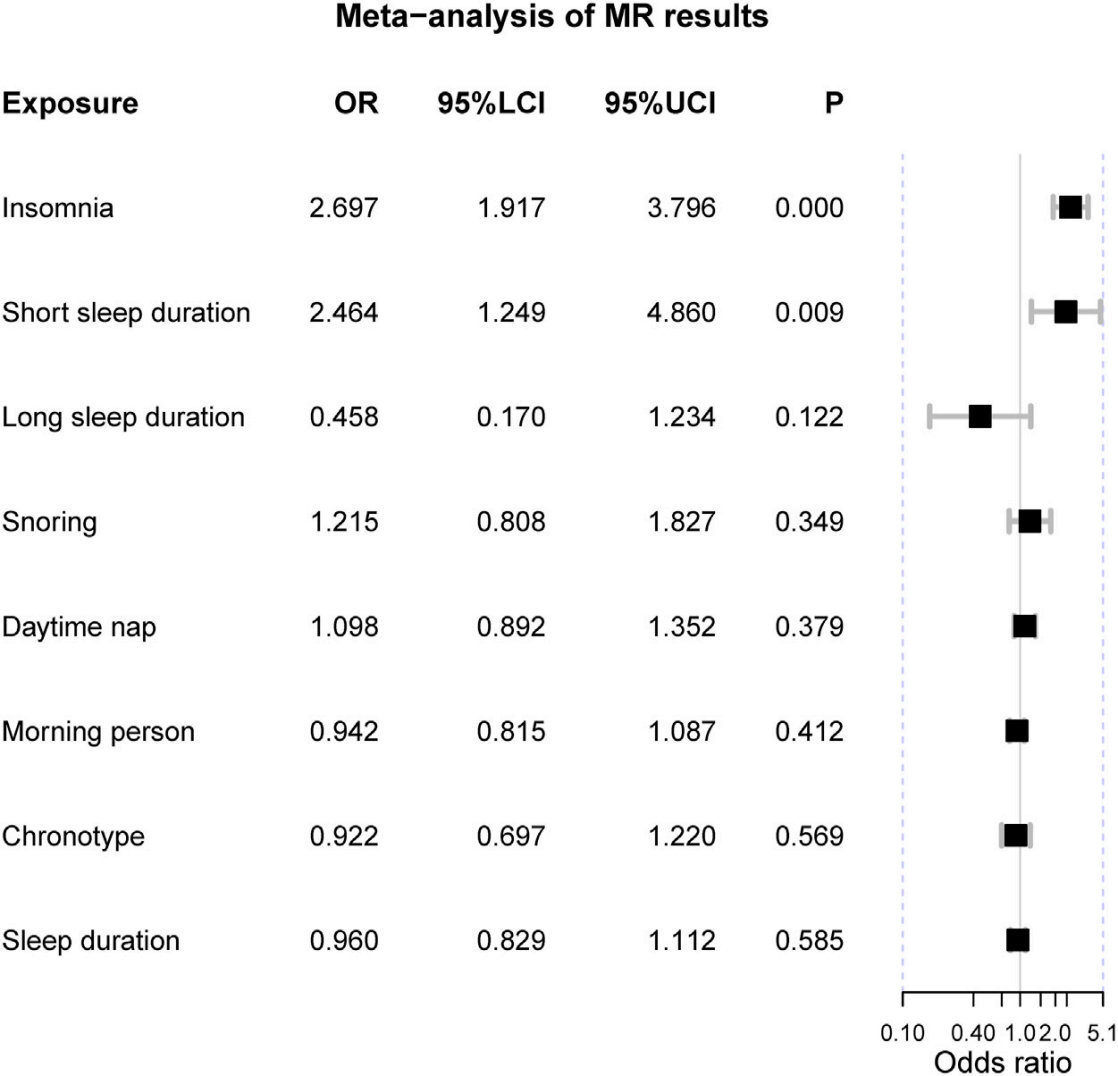
The meta-analysis of Mendelian randomization estimates. Notes: OR = odds ratio; 95%LCI = the lower limit of 95% confidence interval; 95%UCI = the upper limit of 95% confidence interval; P = the *p*-value of OR.

The meta-analysis suggested two genetically-determined sleep exposures can increase the risk of IBS, including insomnia [OR = 2.70 (1.92, 3.80), *p*-value = 1.27 × 10^−8^] and short sleep duration [OR = 2.46 (1.25, 4.86), *p*-value = 0.009] (Figure 3). There was no heterogeneity in these two sleep exposures (I2 = 0% and Q *p*-value > 0.05). As for chronotype and “morning” person, the fixed-effects suggested “morning” chronotype could decrease the risk of IBS [OR = 0.84 (0.79, 0.89), *p*-value = 3.88 × 10^−9^] and so did the “morning” person [OR = 0.90 (0.86, 0.93), *p*-value = 9.32 × 10^−8^]. However, there was obvious heterogeneity in the meta results of the chronotype and “morning” people and their results were insignificant when using the random-effects model. The other sleep-related exposures should not affect the risk of IBS.

The multivariable MR analysis suggested insomnia is an independent risk factor for IBS after adjusting for chronotype [OR = 2.32 (1.57, 3.43), *p*-value = 2.67 × 10^−5^] in the discovery set while not significant in the validation set. After adjusting for short sleep duration, insomnia can elevate the risk of IBS as well [OR = 1.45 (1.13, 1.85), *p*-value = 0.003] in the discovery set but not in the validation set. The statistical powers were all greater than 80% and the bias caused by sample overlapping in the discovery stage was less than 5%, suggesting sufficient power and validity of this MR study. The reverse MR design ruled out the possibility that IBS could increase the risk of insomnia [OR = 0.87 (0.70, 1.08), *p*-value = 0.231].

## Discussion

Briefly, our MR design was focused on the causal relationship between eight different sleep-related exposures and IBS, and the results indicated that insomnia and short sleep duration can increase the risk of IBS, while there is no sufficient evidence to support that the other sleep-related traits have a causal link with IBS, including sleep duration (continuous variable), long sleep duration (binary variable), chronotype, “morning” person, daytime nap, and snoring. Since insomnia is also associated with decreased sleep duration, our results suggest that adequate sleep can increase the risk of developing IBS. In addition, the other null associations also support our conclusion that neither chronotype nor snoring affects the incidence of IBS with adequate sleep duration. Also, excessive sleep duration does not alter the risk of IBS either.

Except for the common pathogenic factors such as genetics, epigenetic changes, and infection, many other complex traits are also risk factors for IBS, including an unhealthy lifestyle and psychological stress (Vasant et al., 2021). In modern times, enormous social and psychological stress drives the development of sleep disorders, especially among middle-aged people (Li et al., 2020). The following relevant studies support our findings that insomnia and short sleep duration may affect pathogenesis and promote the development of IBS through the following physiological mechanisms, such as brain-gut-axis, immune disorders, and circadian rhythms.

Although IBS is a functional gastrointestinal disorder, it was considered that dysfunction within the bidirectional gut-brain axis was significantly associated with IBS (2). IBS is also classified as a disorder of gut-brain interaction since IBS patients often have anxiety and depression, which are also risk factors for IBS in healthy people (Vasant et al., 2021). IBS is thought to often co-occur with mental disorders such as anxiety symptoms and depression, and both of these diseases have a series of biological and psychosocial mechanisms, which are mainly reflected in the gut-brain axis disorders (Staudacher et al., 2021). Meanwhile, sleep behavior is also important to numerous brain functions, including neural cell growth, synaptogenesis, and memory function (Reynolds and O'Hara, 2013; Walker and Stickgold, 2006). Freeman et al. designed a large randomized controlled trial of a psychological intervention for a mental health problem with insomnia, and this study suggested that insomnia is a causal risk factor for the occurrence of mental health problems, and alleviating sleep disorders is particularly important to mental health (Freeman et al., 2017). However, there was a lack of evidence on how insomnia affects the onset of IBS. It should be probable that insomnia might induce intestinal dysfunction via the brain-gut axis since the brain can alter intestinal motility and fluid secretion, intestinal epithelial permeability, and gut microbiota composition (Enck et al., 2016). Besides, insomnia can cause chronic and sustainable stress, which is associated with the onset and exacerbation of IBS (Chang, 2011). Sleep is fundamental to mental health, furthermore, sleep interventions can prevent mental disease and improve psychological health (Freeman et al., 2017; Espie et al., 2019). Interestingly, the largest GWAS study identified six genetic susceptibility loci that were significantly associated with IBS, four of which were located in genes associated with mood and anxiety (NCAM1, CADM2, PHF2/FAM120A, DOCK9) (Eijsbouts et al., 2021). Such results indicated that IBS shared genetic background with insomnia-associated traits. Further genetic analyses with an enlarged sample size should help to identify shared a genetic loci, which can elucidate how insomnia affects the onset of IBS. And psychotherapy for IBS symptoms, especially cognitive behavioral therapy and hypnosis, is helpful for many IBS patients’ symptoms (Chilcot and Moss-Morris, 2013). The accumulating evidence suggests that insomnia can contribute to the increased risk of IBS via mental disorders rising from the gut-brain axis.

To investigate the relationship between sleep restriction and immune function, Circadian et al. recruited nine healthy males to participate in a sleep restriction (4 h of sleep/night for five nights) and sleep recovery protocol (8 h of sleep/night for seven nights), and the results showed that continuous sleep restriction could significantly increase the counts of leukocytes, monocytes, neutrophils, and lymphocytes in peripheral blood, and sleep recovery could partially restore these effects (Lasselin et al., 2015). Furthermore, prolonged periods of wakefulness could increase the soluble tumor necrosis factor-alpha (TNF-alpha) receptor one and interleukin-6 (IL-6) plasma levels in the plasma, which are the messengers connected to the immune and nervous systems (Shearer et al., 2001). In middle-aged and young adults, plasma inflammatory cytokine levels of C-reactive protein (CRP) and IL-6 were significantly elevated in insomnia and short sleep duration populations later after adjustment for confounders, suggesting that clinical interventions targeting sleep disorders might reduce systemic inflammation (Cho et al., 2015). These studies evidenced that sleep insomnia and short sleep duration might play a significant role in contributing to immuno-inflammatory conditions. However, a previous MR study revealed no causal relationship between sleep traits and inflammatory bowel disease (IBD) (Chen et al., 2021). We deemed that IBD is an autoimmune disease that might be largely affected by immune factors, while IBS is a kind of functional intestinal disease that should be affected less. Additionally, the mechanism of autoimmune diseases should be different from that of traditional immune mechanisms, where the immunogen of the former is endogenous while that of the latter is exogenous. Insomnia may affect traditional immune mechanisms, which can affect IBS. Therefore, it is reasonable that insomnia can increase the risk of IBS but not IBD. Meanwhile, high-quality sleep can reduce the incidence rate of infection in both human and animal studies (Ali et al., 2013). In addition, a history of enteric infection is a risk factor for IBS, and patients with a history of enteric infection were more likely to develop IBS than people without a history of acute enteric infection (Marshall et al., 2010; Cremon et al., 2014). Given this close association between sleep disorders and the immune system, we can explain that insomnia is a risk factor for IBS through the disturbance of immunity. However, attention should be paid to the fact that the alteration of immunity in IBS might not be displayed in laboratory examination.

Circadian rhythm refers to the change of life activities in a cycle of 24 h, a regular cycle established by various physiological functions of organisms to adapt to the diurnal variation of the external environment (Patel et al., 2014). Many recent studies have shown that numerous organ systems are related to circadian oscillations, including the kidney, liver, and gut (Vollmers et al., 2012; Firsov and Bonny, 2018; Godinho-Silva et al., 2019). In the circadian disorganization mice model, the permeability of the intestinal epithelial barrier was significantly increased compared with the control group (Summa et al., 2013). Interestingly, PER2, an important gene in regulating the fundamental molecular basis of biological clocks, has also played an essential role in the regulation of colonic motility (Hoogerwerf et al., 2010; Summa et al., 2013). Above all, alterations in sleep mode are more likely to affect the pathogenesis of digestive diseases such as IBS.

The value of sleep quality is underappreciated in clinical medicine. In this MR study, we found that sleep quality plays an important role for us in preventing IBS. The design of our study was rigorous. MR studies were conducted on IBS GWAS data from two different sample sources successively, and a meta-analysis was conducted on the results of the discovery stage and validation stage to ensure the statistical power and reliability of our results. We suggest that the prevention of insomnia may reduce the risk of brain-gut-axis and immunity disorders, guaranteeing a healthy circadian rhythm to prevent and delay the morbidity and progression of IBS. However, several limitations should be pointed out in this study (Ford et al., 2020): horizontal pleiotropy is a natural flaw of MR design though several statistical methods have been applied to avoid it (Vasant et al., 2021); the target population in this study is mainly of European ancestry and the generalizability of our conclusion might not be applied to other ancestries.

## Conclusion

This Mendelian randomization study found insomnia can increase the risk of irritable bowel syndrome, suggesting that improving sleep quality might be effective in improving irritable bowel syndrome.

## Data Availability

Publicly available datasets were analyzed in this study. This data can be found here: https://www.ebi.ac.uk/gwas/.

## Abbreviations

IBS, irritable bowel syndrome; MR, Mendelian randomization; GWAS, genome-wide association study; IV, instrument variable; SNP, single nucleotide polymorphism; IVW, inverse variance weighted; FDR, false discovery rate; CRP, C-reactive protein.

## References

[B1] Ai S. Zhang J. Zhao G. Wang N. Li G. So H. C. (2021). Causal associations of short and long sleep durations with 12 cardiovascular diseases: Linear and nonlinear mendelian randomization analyses in UK biobank. Eur. Heart J. 42 (34), 3349–3357. 10.1093/eurheartj/ehab170 33822910

[B2] Ai S. Z. Dai X. J. (2018). Causal role of rapid-eye-movement sleep on successful memory consolidation of fear extinction. J. Thorac. Dis. 10 (3), 1214–1216. 10.21037/jtd.2018.01.163 29707269 PMC5906322

[B3] Ali T. Choe J. Awab A. Wagener T. L. Orr W. C. (2013). Sleep, immunity and inflammation in gastrointestinal disorders. World J. Gastroenterol. 19 (48), 9231–9239. 10.3748/wjg.v19.i48.9231 24409051 PMC3882397

[B4] Bowden J. Davey Smith G. Haycock P. C. Burgess S. (2016). Consistent estimation in mendelian randomization with some invalid instruments using a weighted median estimator. Genet. Epidemiol. 40 (4), 304–314. 10.1002/gepi.21965 27061298 PMC4849733

[B5] Burgess S. Thompson S. G. (2017). Interpreting findings from Mendelian randomization using the MR-Egger method. Eur. J. Epidemiol. 32 (5), 377–389. 10.1007/s10654-017-0255-x 28527048 PMC5506233

[B6] Campos A. I. García-Marín L. M. Byrne E. M. Martin N. G. Cuéllar-Partida G. Rentería M. E. (2020). Insights into the aetiology of snoring from observational and genetic investigations in the UK Biobank. Nat. Commun. 11 (1), 817. 10.1038/s41467-020-14625-1 32060260 PMC7021827

[B7] Chang L. (2011). The role of stress on physiologic responses and clinical symptoms in irritable bowel syndrome. Gastroenterology 140 (3), 761–765. 10.1053/j.gastro.2011.01.032 21256129 PMC3039211

[B8] Chen L. Yang H. Li H. He C. Yang L. Lv G. (2022). Insights into modifiable risk factors of cholelithiasis: A mendelian randomization study. Hepatol. Baltim. Md) 75 (4), 785–796. 10.1002/hep.32183 PMC9300195 34624136

[B9] Chen M. Peng W. Y. Tang T. C. Zheng H. (2021). Differential sleep traits have No causal effect on inflammatory bowel diseases: A mendelian randomization study. Front. Pharmacol. 12, 763649. 10.3389/fphar.2021.763649 34916940 PMC8669049

[B10] Chilcot J. Moss-Morris R. (2013). Changes in illness-related cognitions rather than distress mediate improvements in irritable bowel syndrome (IBS) symptoms and disability following a brief cognitive behavioural therapy intervention. Behav. Res. Ther. 51 (10), 690–695. 10.1016/j.brat.2013.07.007 23948131

[B11] Cho H. J. Seeman T. E. Kiefe C. I. Lauderdale D. S. Irwin M. R. (2015). Sleep disturbance and longitudinal risk of inflammation: Moderating influences of social integration and social isolation in the Coronary Artery Risk Development in Young Adults (CARDIA) study. Brain Behav. Immun. 46, 319–326. 10.1016/j.bbi.2015.02.023 25733101 PMC4414819

[B12] Cremon C. Stanghellini V. Pallotti F. Fogacci E. Bellacosa L. Morselli-Labate A. M. (2014). Salmonella gastroenteritis during childhood is a risk factor for irritable bowel syndrome in adulthood. Gastroenterology 147 (1), 69–77. 10.1053/j.gastro.2014.03.013 24657623

[B13] Dashti H. S. Daghlas I. Lane J. M. Huang Y. Udler M. S. Wang H. (2021). Genetic determinants of daytime napping and effects on cardiometabolic health. Nat. Commun. 12 (1), 900. 10.1038/s41467-020-20585-3 33568662 PMC7876146

[B14] Dashti H. S. Jones S. E. Wood A. R. Lane J. M. van Hees V. T. Wang H. (2019). Genome-wide association study identifies genetic loci for self-reported habitual sleep duration supported by accelerometer-derived estimates. Nat. Commun. 10 (1), 1100. 10.1038/s41467-019-08917-4 30846698 PMC6405943

[B15] Eijsbouts C. Zheng T. Kennedy N. A. Bonfiglio F. Anderson C. A. Moutsianas L. (2021). Genome-wide analysis of 53, 400 people with irritable bowel syndrome highlights shared genetic pathways with mood and anxiety disorders. Nat. Genet. 53 (11), 1543–1552. 10.1038/s41588-021-00950-8 34741163 PMC8571093

[B16] Emdin C. A. Khera A. V. Kathiresan S. (2017). Mendelian randomization. Mendel. Randomization. Jama. 318 (19), 1925–1926. 10.1001/jama.2017.17219 29164242

[B17] Enck P. Aziz Q. Barbara G. Farmer A. D. Fukudo S. Mayer E. A. (2016). Irritable bowel syndrome. Nat. Rev. Dis. Prim. 2, 16014. 10.1038/nrdp.2016.14 27159638 PMC5001845

[B18] Espie C. A. Emsley R. Kyle S. D. Gordon C. Drake C. L. Siriwardena A. N. (2019). Effect of digital cognitive behavioral therapy for insomnia on health, psychological well-being, and sleep-related quality of life: A randomized clinical trial. JAMA psychiatry 76 (1), 21–30. 10.1001/jamapsychiatry.2018.2745 30264137 PMC6583463

[B19] Firsov D. Bonny O. (2018). Circadian rhythms and the kidney. Nat. Rev. Nephrol. 14 (10), 626–635. 10.1038/s41581-018-0048-9 30143787

[B20] Ford A. C. Sperber A. D. Corsetti M. Camilleri M. (2020). Irritable bowel syndrome. Lancet (London, Engl. 396 (10263), 1675–1688. 10.1016/S0140-6736(20)31548-8 33049223

[B21] Freeman D. Sheaves B. Goodwin G. M. Yu L. M. Nickless A. Harrison P. J. (2017). The effects of improving sleep on mental health (OASIS): A randomised controlled trial with mediation analysis. Lancet. Psychiatry 4 (10), 749–758. 10.1016/S2215-0366(17)30328-0 28888927 PMC5614772

[B22] Godinho-Silva C. Domingues R. G. Rendas M. Raposo B. Ribeiro H. da Silva J. A. (2019). Light-entrained and brain-tuned circadian circuits regulate ILC3s and gut homeostasis. Nature 574 (7777), 254–258. 10.1038/s41586-019-1579-3 31534216 PMC6788927

[B23] Hemani G. Tilling K. Davey Smith G. (2017). Orienting the causal relationship between imprecisely measured traits using GWAS summary data. PLoS Genet. 13 (11), e1007081. 10.1371/journal.pgen.1007081 29149188 PMC5711033

[B24] Hoogerwerf W. A. Shahinian V. B. Cornélissen G. Halberg F. Bostwick J. Timm J. (2010). Rhythmic changes in colonic motility are regulated by period genes. Am. J. Physiol. Gastrointest. Liver Physiol. 298 (2), G143–G150. 10.1152/ajpgi.00402.2009 19926812 PMC2822504

[B25] Jones S. E. Lane J. M. Wood A. R. van Hees V. T. Tyrrell J. Beaumont R. N. (2019). Genome-wide association analyses of chronotype in 697, 828 individuals provides insights into circadian rhythms. Nat. Commun. 10 (1), 343. 10.1038/s41467-018-08259-7 30696823 PMC6351539

[B26] Lane J. M. Jones S. E. Dashti H. S. Wood A. R. Aragam K. G. van Hees V. T. (2019). Biological and clinical insights from genetics of insomnia symptoms. Nat. Genet. 51 (3), 387–393. 10.1038/s41588-019-0361-7 30804566 PMC6415688

[B27] Lasselin J. Rehman J. U. Åkerstedt T. Lekander M. Axelsson J. (2015). Effect of long-term sleep restriction and subsequent recovery sleep on the diurnal rhythms of white blood cell subpopulations. Brain Behav. Immun. 47, 93–99. 10.1016/j.bbi.2014.10.004 25451611

[B28] Li Y. Li G. Liu L. Wu H. (2020). Correlations between mobile phone addiction and anxiety, depression, impulsivity, and poor sleep quality among college students: A systematic review and meta-analysis. J. Behav. Addict. 9 (3), 551–571. 10.1556/2006.2020.00057 32903205 PMC8943681

[B29] Marshall J. K. Thabane M. Garg A. X. Clark W. F. Moayyedi P. Collins S. M. (2010). Eight year prognosis of postinfectious irritable bowel syndrome following waterborne bacterial dysentery. Gut 59 (5), 605–611. 10.1136/gut.2009.202234 20427395

[B30] Orr W. C. Fass R. Sundaram S. S. Scheimann A. O. (2020). The effect of sleep on gastrointestinal functioning in common digestive diseases. Lancet. Gastroenterol. Hepatol. 5 (6), 616–624. 10.1016/S2468-1253(19)30412-1 32416862

[B31] Patel V. R. Eckel-Mahan K. Sassone-Corsi P. Baldi P. (2014). How pervasive are circadian oscillations? Trends Cell Biol. 24 (6), 329–331. 10.1016/j.tcb.2014.04.005 24794425 PMC4732883

[B32] Reynolds C. F. 3rd O'Hara R. (2013). DSM-5 sleep-wake disorders classification: Overview for use in clinical practice. Am. J. Psychiatry 170 (10), 1099–1101. 10.1176/appi.ajp.2013.13010058 24084814

[B33] Rotem A. Y. Sperber A. D. Krugliak P. Freidman B. Tal A. Tarasiuk A. (2003). Polysomnographic and actigraphic evidence of sleep fragmentation in patients with irritable bowel syndrome. Sleep 26 (6), 747–752. 10.1093/sleep/26.6.747 14572130

[B34] Sanderson E. Davey Smith G. Windmeijer F. Bowden J. (2019). An examination of multivariable Mendelian randomization in the single-sample and two-sample summary data settings. Int. J. Epidemiol. 48 (3), 713–727. 10.1093/ije/dyy262 30535378 PMC6734942

[B35] Shearer W. T. Reuben J. M. Mullington J. M. Price N. J. Lee B. N. Smith E. O. (2001). Soluble TNF-alpha receptor 1 and IL-6 plasma levels in humans subjected to the sleep deprivation model of spaceflight. J. Allergy Clin. Immunol. 107 (1), 165–170. 10.1067/mai.2001.112270 11150007

[B36] Song Z. Yang R. Wang W. Huang N. Zhuang Z. Han Y. (2021). Association of healthy lifestyle including a healthy sleep pattern with incident type 2 diabetes mellitus among individuals with hypertension. Cardiovasc. Diabetol. 20 (1), 239. 10.1186/s12933-021-01434-z 34922553 PMC8684653

[B37] Staudacher H. M. Mikocka-Walus A. Ford A. C. (2021). Common mental disorders in irritable bowel syndrome: Pathophysiology, management, and considerations for future randomised controlled trials. Lancet. Gastroenterol. Hepatol. 6 (5), 401–410. 10.1016/S2468-1253(20)30363-0 33587890

[B38] Stern P. (2021). The many benefits of healthy sleep. Sci. (New York, NY) 374 (6567), 550–551. 10.1126/science.abm8113 34709890

[B39] Summa K. C. Voigt R. M. Forsyth C. B. Shaikh M. Cavanaugh K. Tang Y. (2013). Disruption of the circadian clock in mice increases intestinal permeability and promotes alcohol-induced hepatic pathology and inflammation. PLoS One 8 (6), e67102. 10.1371/journal.pone.0067102 23825629 PMC3688973

[B40] Vasant D. H. Paine P. A. Black C. J. Houghton L. A. Everitt H. A. Corsetti M. (2021). British Society of Gastroenterology guidelines on the management of irritable bowel syndrome. Gut 70 (7), 1214–1240. 10.1136/gutjnl-2021-324598 33903147

[B41] Vege S. S. Locke G. R. 3rd Weaver A. L. Farmer S. A. Melton L. J. 3rd Talley N. J. (2004). Functional gastrointestinal disorders among people with sleep disturbances: A population-based study. Mayo Clin. Proc. 79 (12), 1501–1506. 10.4065/79.12.1501 15595333

[B42] Verbanck M. Chen C. Y. Neale B. Do R. (2018). Detection of widespread horizontal pleiotropy in causal relationships inferred from Mendelian randomization between complex traits and diseases. Nat. Genet. 50 (5), 693–698. 10.1038/s41588-018-0099-7 29686387 PMC6083837

[B43] Vollmers C. Schmitz R. J. Nathanson J. Yeo G. Ecker J. R. Panda S. (2012). Circadian oscillations of protein-coding and regulatory RNAs in a highly dynamic mammalian liver epigenome. Cell Metab. 16 (6), 833–845. 10.1016/j.cmet.2012.11.004 23217262 PMC3541940

[B44] Walker M. P. Stickgold R. (2006). Sleep, memory, and plasticity. Annu. Rev. Psychol. 57, 139–166. 10.1146/annurev.psych.56.091103.070307 16318592

[B45] Wang B. Duan R. Duan L. (2018). Prevalence of sleep disorder in irritable bowel syndrome: A systematic review with meta-analysis. Saudi J. Gastroenterol. 24 (3), 141–150. 10.4103/sjg.SJG_603_17 29652034 PMC5985632

